# Metamaterial enhanced sensor for powder material classification

**DOI:** 10.1038/s41598-024-71175-y

**Published:** 2024-09-12

**Authors:** Hany M. Zamel, Sherine Ismail Abd El-Rahman, Ahmed M. Attiya

**Affiliations:** Microwave Engineering Department, Electronics Research Institute (ERI), Cairo, 11843 Egypt

**Keywords:** Engineering, Materials science

## Abstract

In this paper, a simple and efficient approach is presented to classify different power materials based on a one port microwave sensor in X-band. This classification focuses on powder materials, unlike prior studies that focused on liquids (castor oil, neem oil, sunflower oil, sesame oil, and mahua oil), this classification represents a shift towards powdered materials. The response of the proposed sensor is enhanced by adding a metamaterial (MTM) unit cell of F-shape to focus electromagnetic waves on the sample under test. This metamaterial-based sensor is designed to differentiate between different types of materials based on the corresponding reflection coefficient. The sample under test is included inside a dielectric box inserted inside a rectangular waveguide. The MTM unit cell is added on the front face of this box towards the direction of the incident wave. The resonance frequency depends on the characteristics of the powder material inside the box. The MTM unit cell enhances this resonance to simplify the process of classification of different materials. The measured results show that the proposed sensor can detect a wide range of powder materials, including clay, cement, sand, and mixtures: cement & sand, and clay & sand. The designed sensor can be used in various applications, including detection and classification of different powder materials in industrial applications.

## Introduction

Metamaterials are synthetic materials with electromagnetic properties that do not exist in nature. The physical properties of MTMs are highly dependent on the shape, design, orientation, and dimensions of their unit cells. Metamaterials are good candidates for focusing electromagnetic waves in near field region. This property makes metamaterials to be suitable for sensing applications. On the other hand, different techniques were introduced material classifications based on their electromagnetic properties at high frequencies like cavity perturbation method^1^ free-space method^2,3^, planar resonator method^4^ and transmission-line method^5^.

Recently, metamaterial-based sensors were developed to classify different materials for different applications^6–9^. This technique is mainly based on the shift in the measured resonance frequency for these materials. Romera et al. presented a technique for measuring the thickness and relative permittivity of solid and liquid materials by using two split-ring resonator (SRR) in a frequency range less than 2 GHz^10^. The sensing process in this case is based on notches in the corresponding transmission coefficients. Abdulkarim et al. presented another metamaterial sensor for the detection of liquid chemicals^11^. Different liquids including clean and waste transformer oil, corn oil, cotton oil and olive oil were classified by using this method. A reflected mirror rectangular split-ring resonator-shaped metamaterial sensor is also designed for detecting type and thickness of different materials^12^. Tumkaya et al. introduced a microwave sensor for distinguishing between branded and unbranded fuel samples^13,14^. Another metamaterial sensor based on adjacent triple circle SRR shaped within the X band is introduced for the identification of various oils, fluids, and chemicals using microwave frequencies. This MTM sensor exhibits a good quality factor and high sensitivity in both frequency shifts and amplitude variations^15^.

Islam et al. presented a tri-circle SRR metamaterial sensor, specifically designed for X-band detection and classification of different liquids, including coconut, olive, clean, sunflower and canola oils. This sensor achieves real-time, reliable, and specific recognition of various samples^16^. An oval-shaped MTM sensor for evaluating glucose levels in watery mixtures is also presented in Ref.^17^. Its impressive sensitivity of 0.037 GHz per 30 mg/dL of glucose solution makes it a powerful tool for glucose detection. Euclides et al., presented a microwave sensor for liquid dielectric characterization based on MTM complementary SRR at 2.4 GHz^18^. The liquid sample fills a glass capillary tube, inserted into a hole within the center of the complementary SRR. This hole is designed to match the capillary's external diameter, ensuring optimal interaction with both the CSRR and the surrounding transmission line. A flexible double-sided microwave sensor based on nested-complementary split ring resonator loaded U-shaped microstrip structure for dielectric constant monitoring of liquids is presented by Jianbing et al.^19^. A material detection sensor utilizing a Substrate Integrated Waveguide (SIW) cavity resonator is introduced in Ref.^20^. Three conductive vias define the three electric walls of the SIW cavity resonator, while the fourth wall is realized by a quarter-wavelength stub, influencing the modal characteristics and electromagnetic field distribution. Partial removal of the dielectric substrate creates a cavity below the stub, forming a sensitive region for sample interaction. Introducing a liquid or material within this cavity modifies the effective permittivity experienced by the stub, leading to a shift in the cavity resonator's resonant frequency due to altered modal characteristics.

Zhang et al. introduced a microwave metamaterial absorber specifically designed for non-destructive sensing in the grain industry, potentially improving efficiency and minimizing waste within the agricultural and food sectors^21^. Islam et al. developed a metamaterial sensor using a unique star-enclosed split ring resonator design. This sensor effectively detects adulteration in gasoline and oil^22^. The research by Yu et al. explores a new application to act as sensors for terahertz metamaterial absorbers. This dual functionality is particularly attractive for compact or integrated systems^23^. The liquid or solid sample under test (SUTs) can then be placed in the container, which is sandwiched between the chiral metamaterial structures. Alternatively, the SUTs can be positioned at the front or back of the metamaterial sensing surface. This can be achieved by placing the SUTs in a waveguide adjuster, which serves as a holder for the SUTs^24^. A star-shaped, dual-band (C and X band) tunable microwave sensor designed for high-performance sensing of both solid and liquid materials, including FR-4 substrates, Rogers laminates (RO3035, RO5880, RT6202), and various liquids (milk, oil, water, and even an energy drink). The metamaterial array and its liquid reservoir positioned between the waveguide structures, eliminating the need for complete enclosure within the waveguide cavity^25^. A key feature of waveguide sensor topology is the use of chiral metamaterials. These materials strategically arrange resonators in an asymmetric manner, leading to unique benefits for sensor performance^26^. To achieve negative refraction more efficiently, the design utilizes resonators with chiral asymmetry. These resonators reside on opposing sides of the substrate and exhibit a geometry that prevents perfect mirroring. This configuration offers additional advantages through mutual coupling effects^27^. One example of this concept is the use of split-ring resonators (SRRs) in these double-sided chiral structures^28^.

MTM sensors have emerged applications in various fields, including biological and medical diagnostics, agricultural monitoring, and environmental sensing^29–31^. This paper presents a sensor based on one-port waveguide with a modified F-shape unit cell to classify different powder in X band. The paper is organized as follows: Section “Design of metamaterial unit cell sensor” introduces the unit cell sensor design. Section “Equivalent circuit of the F-shape unit cell sensor” equivalent circuit and field distribution. Section “Analysis and design of F-shape sensor inside WG” operation of one-port waveguide sensors with MTM unit cell. Finally, conclusion is presented in Section “Conclusion”.

## Design of metamaterial unit cell sensor

**Fig. 1:**
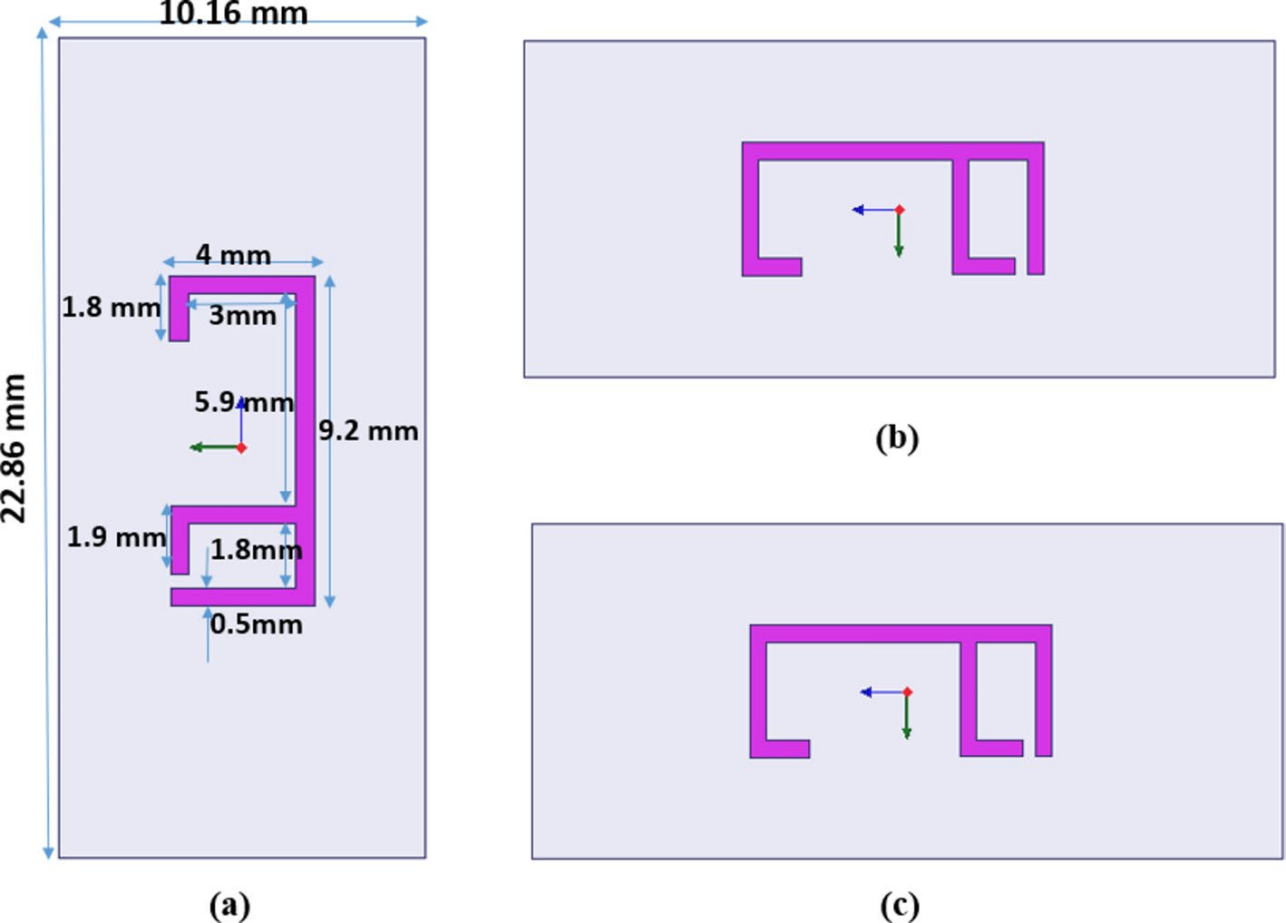
(**a**) Geometry of the proposed antenna, (**b**) top, (**c**) bottom.

**Fig. 2:**
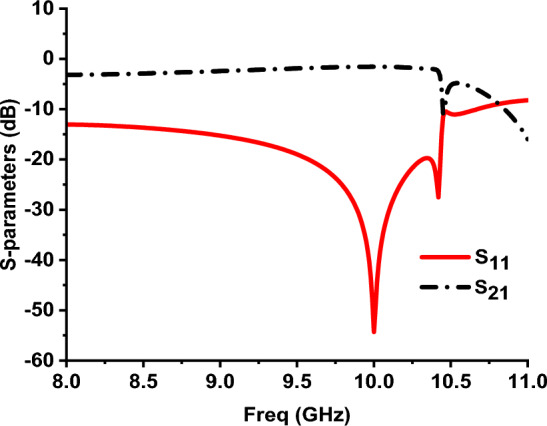
Simulated reflection and transmission coefficients for the proposed MTM structure.

The numerical studies of the F-shaped resonator are designed and analyzed using finite element method (FEM) based HFSS software. The proposed sensor design operates in the X-band frequency regime. The dimensions of unit cell are adjusted to fill the waveguide section WR-90 with inner dimensions 22.86 × 10.16 mm^2^, as shown in Fig. 1. This size selection facilitates compatibility with an X-band waveguide for experimental work using the waveguide measurement method. To determine the optimal dimensions for the proposed unit cell sensor, a parametric analysis is performed to be suitable for the analysis in the frequency region from 9 to 11 GHz. The top and the bottom layers of the MTM unit cell are illustrated in Fig. 1b and c. This unit cell is printed on FR4 dielectric substrate with a dielectric constant ${\varepsilon }_{r}=$ 4.3 and loss tangent tan $\delta =$ 0.02. The substrate thickness is 1.6 mm. Figure 2, shows the reflection and transmission coefficients of this unit cell.

The equivalent metamaterial characteristics of this unit cell can be obtained by using the resulting complex reflection and transmission coefficients as follows^32^:

1 $$n= \frac{1}{kd}{\text{cos}}^{-1}\left[\frac{1}{2{S}_{21}}\left(1-{S}_{11}^{2}+{S}_{21}^{2}\right)\right]$$

2 $$z=\sqrt{\frac{{\left(1+{S}_{11}\right)}^{2}-{{S}_{21}}^{2}}{{\left(1-{S}_{11}\right)}^{2}-{{S}_{21}}^{2}}}$$

3 $$\varepsilon = \frac{n}{z}$$

4 $$\mu ={n}^{2}/\varepsilon $$

**Fig. 3:**
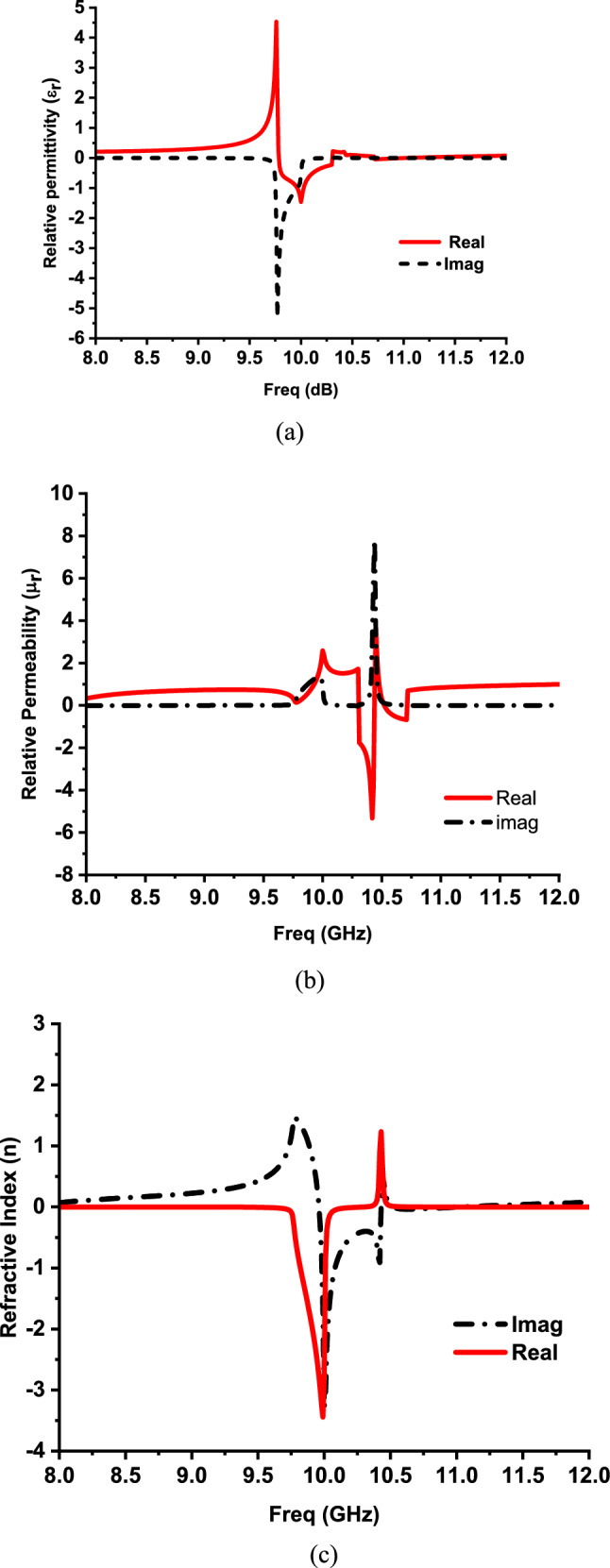
(**a**) Relative permittivity for the proposed MM structure (**b**) Relative permeability for the proposed MM structure (**c**) Refractive index for the proposed MM structure.

The effective parameters (ε, μ, n and z) are shown in Fig. 3a–c. It can be noted that negative real permittivity for this unit cell lies in the frequency ranges from 9.785 GHz to 10.3 GHz and 10.66 GHz to 10.96 GHz. On the other hand, negative real permeability lies in the frequency ranges from 10.3 GHz to 10.44 GHz and 10.52 GHz to 10.71 GHz. Negative real refractive index is observed over the frequency ranges from 9.96 GHz to 10.43 GHz and 10.55 GHz to 10.95 GHz.

## Equivalent circuit of the F-shape unit cell sensor

**Fig. 4:**
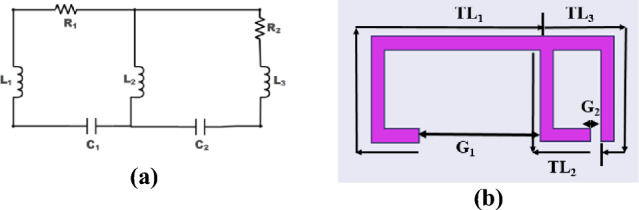
Equivalent circuit of the F-shape unit cell sensor.

The equivalent circuit of the designed F-shape unit cell sensor is shown in Fig. 4a. The unit cell can be divided into five parts such as TL1, TL2, TL3, G1, and G2 as shown in Fig. 4b.

Transmission lines inherently introduce inductive effects due to their physical characteristics. These effects can be defined as L1, L2, L3 respectively. The presence of a gap is equivalent to C1 and C2 which correspond to the capacitive coupling between the conductors. The unit cell's behavior can be accurately modeled using an RLC circuit, as illustrated in Fig. 4a.

The impedance of the F-shape unit cell sensor is defined as Refs.^33,34^

5 $${Z}_{t}= {R}_{t }+j\omega {L}_{t}+\frac{1}{j\omega {C}_{t}}$$

where ${Z}_{t}$, ${R}_{t}$, ${L}_{t}$, ${C}_{t}$ are respectively denoting the total impedance, total resistance, total inductance and total capacitance of the unit cell structure. The resonance frequency is given by Refs.^33,34^

6 $${f}_{r}= \frac{1}{2\pi \sqrt{{L}_{t}{C}_{t}}}$$

**Fig. 5:**
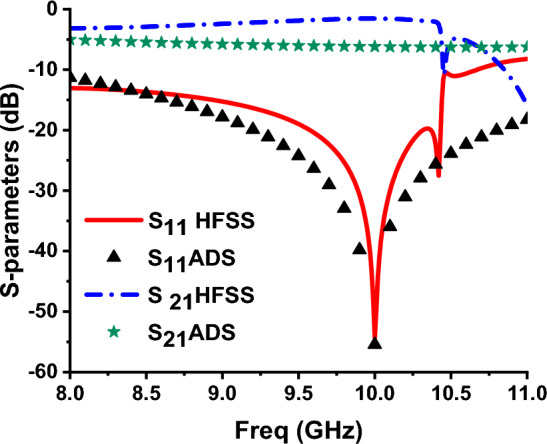
Comparison of unit cell’s reflection coefficient using ADS and HFSS.

The performance of the unit cell by using HFSS and the corresponding equivalent circuit by using ADS is shown in Fig. 5. Table 1, presents the different components values of the equivalent circuit.

**Table 1:** Values of different components of the Equivalent circuit.

Component	L_1_	L_2_	L_3_	R_1_	R_2_	C_1_	C_2_
Value	24.695 nH	7.491 nH	0.425 nH	32.75 Ω	26.075 Ω	0.05 pF	0.518 pF

## Analysis and design of F-shape sensor inside WG

**Fig. 6:**
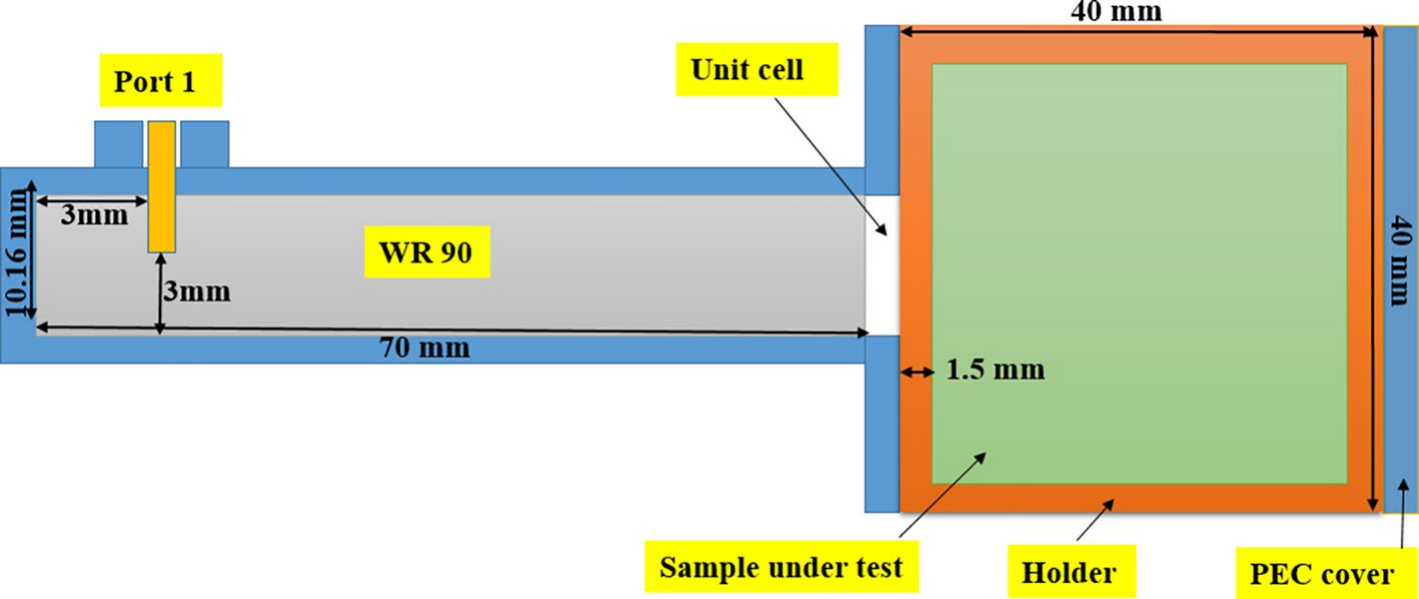
System diagram of the microwave sensing process.

Figure 6 shows the configuration of the proposed sensor for classifying powder materials. It consists of a unit cell of metamaterial embedded inside on the open end of a rectangular waveguide. The powder material is placed in dielectric container attached to the open end of the waveguide. The sample container is a hollow cube of outer length 40 mm and thickness 1.5 mm made by using a material of ε_r_ = 3.6. The basic theory of the present sensor is that the metamaterial unit cell is used to focus the fields inside the sample container to enhance the reflection sensitivity measured by the open-ended waveguide section. The dielectric properties of the material under test changes the resonant frequency of the reflection coefficient. This change in resonant frequency is used to classify different materials^33^. The container is filled with different powder materials under test and is hold attached to the waveguide as shown in Fig. 6. The reflection coefficient is measured in the frequency range from 9 to 11 GHz. The dielectric constant (${\varepsilon }_{r}$) and loss tangent (tan $\delta$) of different samples are previously obtained experimentally by using Dielectric Assessment Kite (DAK) are listed in Table 2. These values are used in the present study to introduce the simulation results for the proposed sensor to discriminate between these different materials. These simulation results are verified experimentally to introduce the applicability of this sensor for fast discrimination between these materials. The proposed materials are mainly used in civil engineering. Thus, it is expected that this simple sensor can be quite useful for onsite tests for civil engineering.

**Table 2:** Measured average dielectric constant (${\varepsilon }_{r}$) and Tan $\delta$ of powder materials under test.

	Cement	Sand	Clay	Sand with clay (Mixing ratio 50%)	Sand with cement (Mixing ratio 50%)
${\varepsilon }_{r}$	2.7	1.78	3.3	6.5	2.1
tan $\delta$	0.19	0.23	0.28	0.24	0.13

**Fig. 7:**
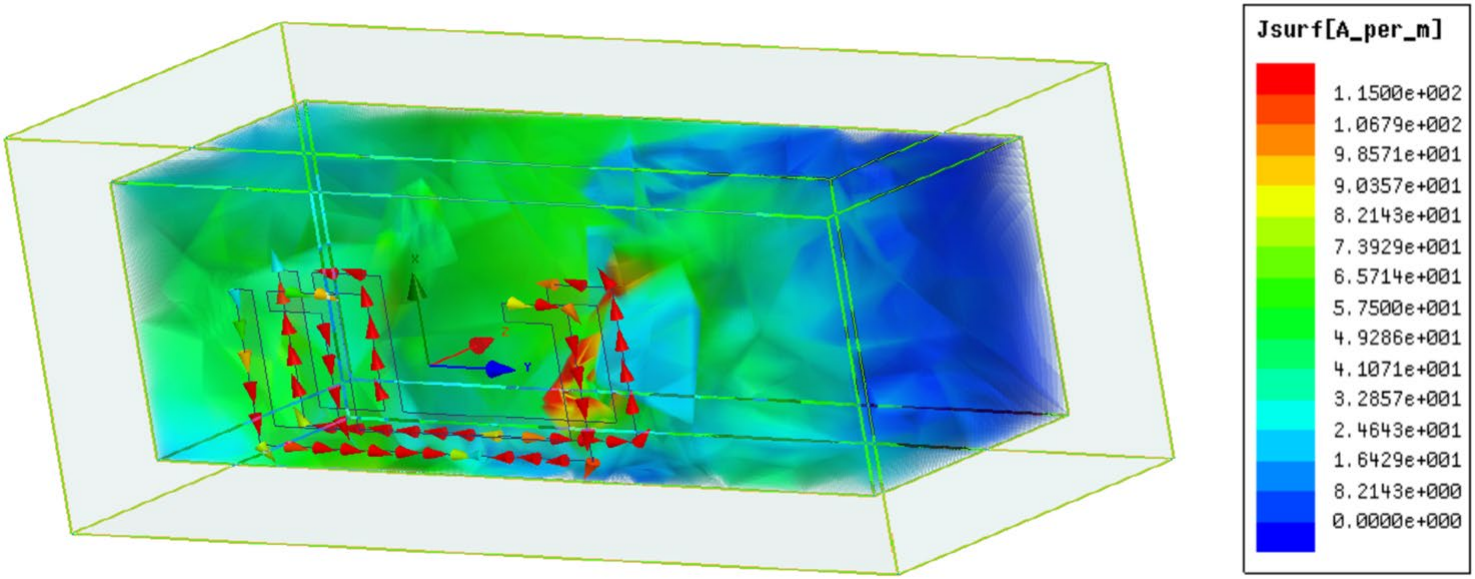
Surface current distribution for the proposed design.

**Fig. 8:**
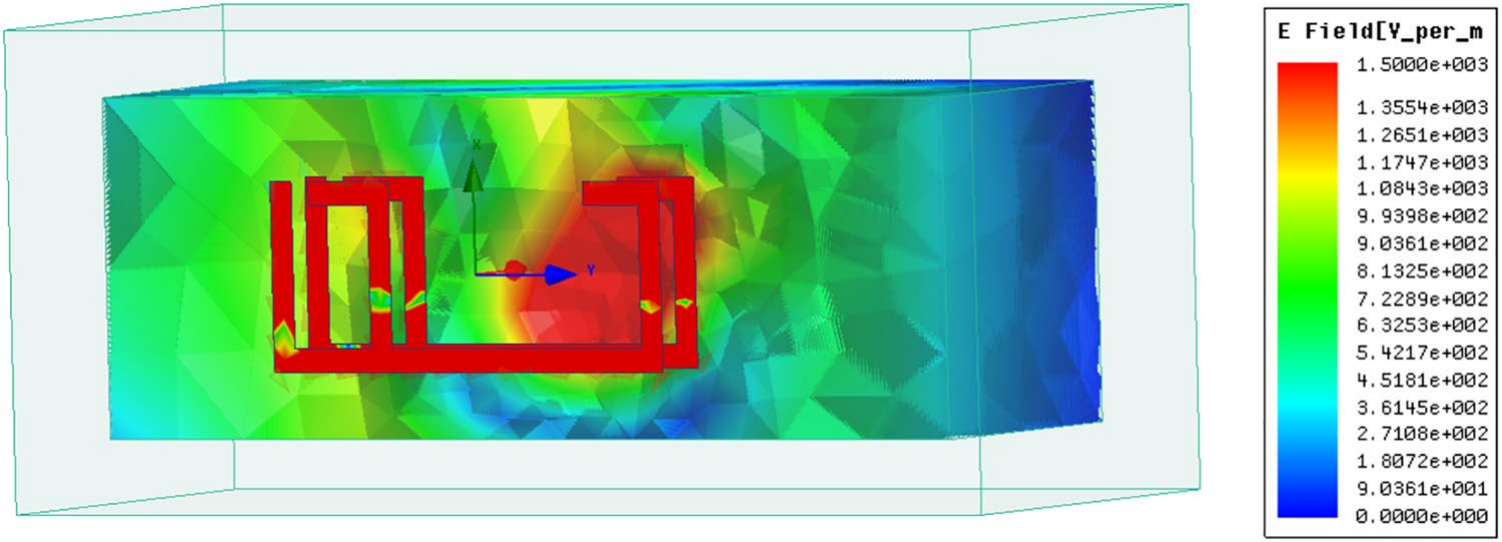
Electric field distribution for the F-shape sensor.

Figures 7 and 8 illustrate, respectively, the electric field and surface current distributions within the sand sample at the resonant frequency of 10.48 GHz. Figure 7 highlights how, for air, the electric field primarily concentrates near the resonator's surface. While electromagnetic fields can travel through conductive cables, the field within the sensor is expected to be in the Transverse Electromagnetic (TEM) mode. This mode dictates that the electric field component is zero. Analyzing the distributions of both the electric field and surface current is crucial to comprehend the underlying theory of the sensor's design. These distributions offer insights into the energy storage and loss mechanisms within the device, as changes in these fields reveal information about the contained energy and any energy dissipation.

The distribution of surface currents flowing along the edges of the F-shaped sensor at its resonant frequency of 10.48 GHz (when a sand sample is present), as shown in Fig. 8. Red arrows visualize the direction of current flow. The currents are strongest and most concentrated at the top and bottom of the sensor, flowing in clockwise and counter-clockwise directions.

**Fig. 9:**
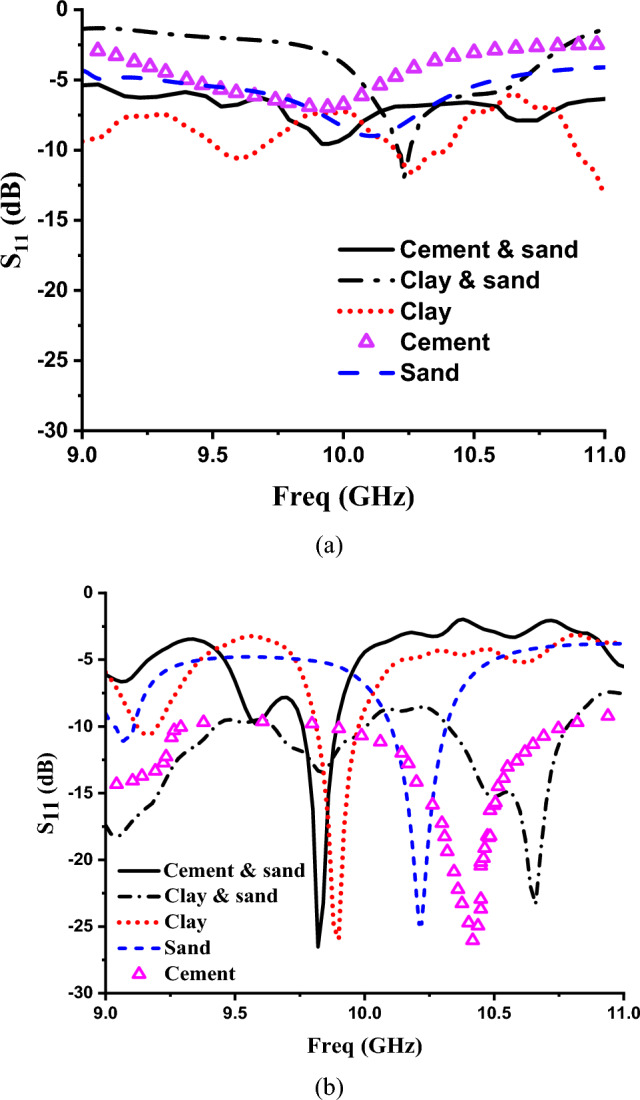
Simulated reflection coefficient of the designed sensor for different materials (**a**) without MTM unit cell (**b**) with MTM unit cell.

Figure 9, shows the reflection coefficient for these different powder samples without the MTM unit cell and with MTM unit cell. It can be noted that the MTM unit cell enhances the discrimination between different samples by introducing sharp clear different resonances for these different materials. The obtained resonance frequencies with MTM are 9.82 GHz, 9.92 GHz and 10.22 GHz for the cement with sand, clay and sand with − 26.53 dB, − 25.95 dB and -24.78 dB magnitude, respectively. Whereas, for cement and clay with sand, the magnitude values are − 26.03 dB at 10.42 GHz and − 23.33 dB at 10.66 GHz, respectively.

## Experimental verification

**Fig. 10:**
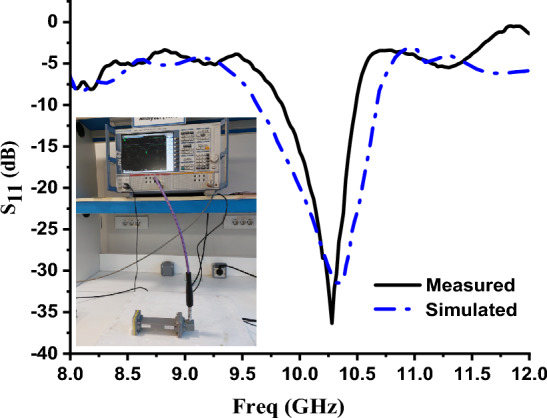
Measured and simulated reflection coefficient of the MTM unit cell inside WG Section.

The measured and simulated reflection coefficient for the proposed unit cell inside WG is shown in Fig. 10. It can be noted that there is a good agreement between the measured and simulated results. However, little fabrication and calibration errors introduce minor discrepancies between them. Significant noise observed in the results, due to fabrication and calibration tolerances produce some minor performance variations.

**Fig. 11:**
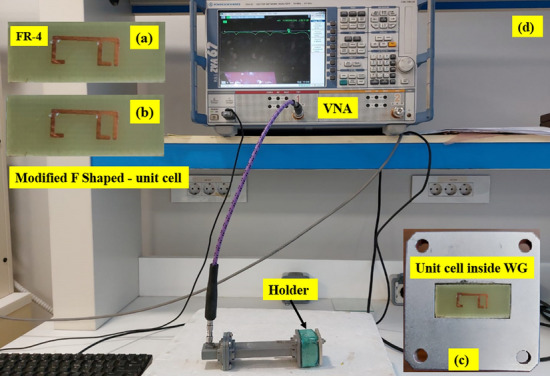
(**a**) Front view of the fabricated metamaterial-based sensor, (**b**) back view, (**c**) Unit cell inside waveguide and (**d**) experimental setup with waveguide configuration.

The proposed setup for powder material classification is shown in Fig. 11. When designing an effective sensor to distinguish among powder samples, the frequency band should be carefully determined because the dielectric permittivity depends on the frequency. The network analyzer and dielectric probe kit which are used to measure the dielectric permittivity of the samples are adjusted to be in the frequency range from 8 to12 GHz.

During the measurements, certain oscillatory fluctuations were detected on the VNA. These fluctuations are attributed to the combined effects of waveguide coupling and MTM sensor fabrication tolerances within the prototype FR-4 substrate layer. Fluctuations were additionally influenced by the dielectric properties of the substrate and environmental factors affecting the measurement.

**Fig. 12:**
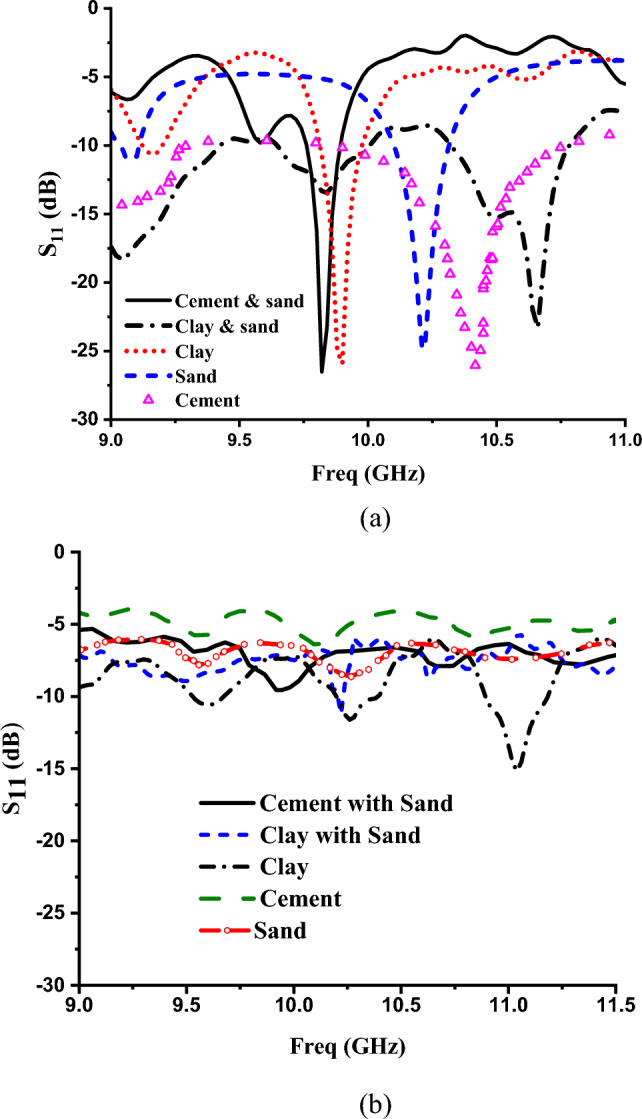
Measured S11 response of powder samples (**a**) with MTM structure (**b**) without MTM structure.

Figure 12 illustrates the simulated and experimental results for reflection coefficients of different powder samples. It can be noted that experimental results align with the numerical study. It is concluded that from these figures without MTM structure, there were no transition in the resonance frequency for different powder samples which leads to difficulty in the classification process. On the other hand, using MTM unit cell introduces different resonance frequencies for powder samples. This can be explained due to the field enhancement by the MTM unit cell which acts as a lens for electromagnetic waves, focusing them onto the sample under test. This concentrated energy makes it easier to distinguish between different samples based on their interactions with the incident waves.

Table 3 shows the responses of reflection coefficients for different powder samples with/without the MTM structure.

**Table 3:** Comparison for different samples with/without the MTM structure.

Type of specimens	With metamaterial				Without metamaterial			
	Resonance frequency (GHz)		S_11_ (dB)		Resonance frequency (GHz)		S_11_ (dB)	
	Meas	Sim	Meas	Sim	Meas	Sim	Meas	Sim
Cement and sand	9.86	9.82	− 22.17	− 26.53	10.03	9.92	− 8.89	− 9.56
Clay and sand	10.69	10.66	− 21.77	− 23.33	10.24	10.23	− 11.45	− 12.04
Clay	9.81	9.92	− 27.08	− 25.95	9.58	9.47	− 10.55	− 14.49
Cement	10.16	10.42	− 25.29	− 26.04	10.09	9.94	− 6.38	− 6.95
Sand	10.34	10.22	− 26.04	− 24.79	10.27	10.11	− 8.618	− 8.99

### Comparison with other related works

The proposed sensor performance metrics, including frequency band, material under test, sensing parameters, and resonant frequency shift, are compared with those of established microwave sensors documented in the literature, as shown in Table 4. Unlike other microwave sensors which are developed to measure liquid materials based on both S11 and/or S21 response, the present sensor is used to classify powder samples by analyzing S11 only with frequency shifts ranging from 30 to 60 MHz between different samples.

**Table 4:** Comparative evaluation of performance Metrics: Our Work versus previous work.

Ref.	Frequency band (GHz)	Material under test	Sensing parameters	Resonant frequency shift (MHz)
^11^	8–12	oils (clean, waste transformer, corn, cotton and olive), Diesel (branded and unbranded)	S_21_	50–250
^15^	9–10	Oils (olive, corn sunflower and palm), Fluids (clean and waste brake), Benzene and carbon-tetrachloride chemicals	S_11_	70–100
^16^	8–12	Oils (coconut, olive, sunflower, canola, clean and waste engine)	S_21_	170–210
^33^	8.5 – 10.5	Gasoline (authentic and inauthentic), Diesel (authentic and inauthentic), oil (clean and waste)	S_21_	70–350
^35^	8 -12	Oils (castor, neem, sunflower, sesame and mahua)	S_11_ and S_21_	30–90
This work	9–11	Powder (clay, cement, and sand), Mixtures (cement & sand, and clay & sand)	S_11_	30–60

## Conclusion

This paper presents a modified F-shaped unit cell sensor for the classification of various powder materials based on a one-port waveguide measurement. The classification performance of various powder samples was compared with and without the use of an MTM structure by using HFSS. The frequency-shifting property allows the sensor to achieve high sensitivity in distinguishing between different samples. The dimensions of the designed sensor are 22.86 × 10.16 mm^2^, making it perfectly compatible with X-band waveguide dimensions. The equivalent circuit of the F-shape unit cell sensor was studied by using ADS. The proposed sensor utilizes frequency shifting to achieve precise discrimination between various samples. The simulation and experimental results demonstrate that the presence of an MTM structure significantly enhances the classification accuracy for different powder samples. The recommended sensor can be used in various applications, including the detection of powder materials and industrial applications.

## Data Availability

The datasets used and/or analysed during the current study available from the corresponding author on reasonable request.

## References

[1] Yu, C. *et al.* An improved cavity-perturbation approach for simultaneously measuring the permittivity and permeability of magneto-dielectric materials in Sub-6G. *IEEE Access* **9**, 14807–14815 (2021). 10.1109/ACCESS.2021.3052874

[2] Hasar, U. *et al.* Improved method for permittivity determination of dielectric samples by free-space measurements. *IEEE Trans. Inst. Meas.* **71**, 1–8 (2022).

[3] Chu, W., Dan, S., You-gang, G. & Ming Z. A new free-space method for the permittivity measurement of walls in indoor environments. Int. Sym. Elec. Comp. (EMC) 1–4 (2017).

[4] Bahar, A., Zakaria, Z., Rashid, S., Isa, A., Ruslan, E., Alahnomi, R. Microstrip planar resonator sensors for accurate dielectric measurement of microfluidic solutions. Int. Conf. Electron. Des. 416–421 (2017).

[5] Piekarz, I., Sorocki, J., Wincza, K. & Gruszczynski, S. Liquids permittivity measurement using two-wire transmission line sensor. *IEEE Sensor* **18**, 7458–7466 (2018). 10.1109/JSEN.2018.2856889

[6] Mayani, M., Martínez, F., Domingo, J. & Giannetti, R. Resonator-based microwave metamaterial sensors for instrumentation: Survey, classification, and performance comparison. *IEEE Trans. Inst. Meas.* **70**, 1–14 (2021). 10.1109/TIM.2020.3040484

[7] Tamera, A. *et al.* Metamaterial based sensor integrating transmission line for detection of branded and unbranded diesel fuel. *Chem. Phys. Lett.* **742**, 1–7 (2020).

[8] Bakır, M. *et al.* Metamaterial sensor for transformer oil and microfluidics. *Aces J.* **34**, 799–806 (2019).

[9] Altintas, O., Aksoy, M., Unal, E. & Karaaslan, M. Chemical liquid and transformer oil condition sensor based on metamaterial-inspired labyrinth resonator. *J. Electrochem. Soc.* **166**, B482–B488 (2019). 10.1149/2.1101906jes

[10] Romera, G., Martínez, F., Gil, M., Martínez, J. & Vargas, D. Submersible printed split-ring resonator-based sensor for thin-film detection and permittivity characterization. *IEEE Sensors J.* **16**, 3587–3596 (2016). 10.1109/JSEN.2016.2538086

[11] Abdulkarima, I. *et al.* Design and study of a metamaterial-based sensor for the application of liquid chemicals detection. *J. Mater. Res. Technol.* **9**, 10291–10304 (2020). 10.1016/j.jmrt.2020.07.034

[12] Islam, Md. *et al.* Metamaterial sensor based on reflected mirror rectangular split ring resonator for the application of microwave sensing. *Measurement* **198**, 1–11 (2022).

[13] Tümkaya, A., Unal, E. & Sabah, C. Metamaterial-based fuel sensor application with three rhombus slots. *Int. J. Modern Phys. B* **33**, 1950276 (2019). 10.1142/S021797921950276X

[14] Bakır, M. *et al.* A comprehensive study on fuel adulteration sensing by using triple ring resonator type metamaterial. *J. Electrochem. Soc.* **166**, B1044–B1052 (2019). 10.1149/2.1491912jes

[15] Islam, Md. *et al.* Metamaterial sensor based on rectangular enclosed adjacent triple circle split ring resonator with good quality factor for microwave sensing application. *Sci. Rep.* **12**, 1–18 (2022). 35474227 10.1038/s41598-022-10729-4 PMC9042823

[16] Islam, R. *et al.* Tri circle split ring resonator shaped metamaterial with mathematical modeling for oil concentration sensing. *IEEE Access* **9**, 161087–161102 (2021). 10.1109/ACCESS.2021.3131905

[17] Islam, M., Hoque, A., Almutairi, F. & Amin, N. Left-handed metamaterial-inspired unit cell for S-band glucose sensing application. *Sensors* **19**, 1–12 (2019). 10.3390/s19010169 PMC6339072 30621259

[18] Chuma, E., Iano, Y., Fontgalland, G. & Roger, L. Microwave sensor for liquid dielectric characterization based on metamaterial complementary split ring resonator. *IEEE Sensor J.* **18**, 9978–9983 (2018). 10.1109/JSEN.2018.2872859

[19] Xie, J., Chen, J., Li, Z. & Yuan, W. Flexible microwave sensor films based on nested-complementary split ring resonator for liquid dielectric constant detection. *Sensors Actuators A Phys.* **359**, 1–11 (2023). 10.1016/j.sna.2023.114461

[20] Soltan, A., Sadeghzadeh, A. & Nezhad, S. Microwave sensor for liquid classification and permittivity estimation of dielectric materials. *Sensor Actuators* **336**, 113397 (2022). 10.1016/j.sna.2022.113397

[21] Zhang, Y., Zhao, J., Cao, J. & Mao, B. Microwave metamaterial absorber for nondestructive sensing applications of grain. *Sensors* **18**, 1–10 (2018). 10.3390/s18061912 PMC6021792 29895793

[22] Islam, Md. *et al.* Star enclosed circle split ring resonator-based metamaterial sensor for fuel and oil adulteration detection. *Alex. Eng. J.* **67**, 547–563 (2023). 10.1016/j.aej.2023.01.001

[23] Yu, J., Lang, T. & Chen, H. All-metal terahertz metamaterial absorber and refractive index sensing performance. *Photonics* **8**, 1–8 (2021). 10.3390/photonics8050164

[24] Rabbani, M. *et al.* Orthogonal centre ring field optimization triple-band metamaterial absorber with sensing application. *Eng. Sci. Technol. Int. J.* **49**, 1–16 (2024).

[25] Chowdhury, M., Islam, M., Alzamil, A., Soliman, M. & Samsuzzaman, M. A tunable star-shaped highly sensitive microwave sensor for solid and liquid sensing. *Alex. Eng. J.* **86**, 644–662 (2024). 10.1016/j.aej.2023.12.001

[26] Ma, X. *et al.* Meta-chirality: Fundamentals, construction and applications. *Nanomaterials* **7**, 1–17 (2017). 10.3390/nano7050116 PMC5449997 28513560

[27] Wang, B., Zhou, J., Koschny, T., Kafesaki, M. & Soukoulis, C. Chiral metamaterials: Simulations and experiments. *J. Opt. A Pure Appl.* **11**, 1–10 (2009).

[28] Ekmekci, E., Kose, U., Cinar, A., Ertan, O. & Ekmekci, Z. The use of metamaterial type double-sided resonator structures in humidity and concentration sensing applications. *Sens. Actuators A Phys.* **297**, 1–15 (2019). 10.1016/j.sna.2019.111559

[29] Ebrahimi, A., Tovar-Lopez, F., Scott, J. & Ghorbani, K. Differential microwave sensor for characterization of glycerol–water solutions. *Sensors Actuators B Chem.* **321**, 1–8 (2020). 10.1016/j.snb.2020.128561

[30] Velez, P., Martin, F., Fernandez-Garcia, R. & Gil, I. Embroidered textile frequency splitting sensor based on stepped-impedance resonators. *IEEE Sensor* **22**, 8596–8603 (2022). 10.1109/JSEN.2022.3163165

[31] Ebrahimi, A. *et al.* Highly sensitive phase-variation dielectric constant sensor based on a capacitively-loaded slow-wave transmission line. *IEEE Trans. Circuits Syst.* **68**, 2787–2799 (2021). 10.1109/TCSI.2021.3074570

[32] Smith, D., Vier, D., Koschny, T. & Soukoulis, C. Electromagnetic parameter retrieval from inhomogeneous metamaterials. *Phys. Rev. E* **71**, 1–11 (2005). 10.1103/PhysRevE.71.036617 15903615

[33] Altıntas, O., Aksoy, M. & Unal, E. Design of a metamaterial inspired omega shaped resonator-based sensor for industrial implementations. *Phys. E Low-Dimen. Syst. Nanostruct.* **116**, 1–10 (2020). 10.1016/j.physe.2019.113734

[34] Haq, T. & Koziel, S. Novel complementary resonator for dielectric characterization of substrates based on permittivity and thickness. *IEEE Sensors J.* **24**, 195–203 (2024). 10.1109/JSEN.2023.3332124 PMC10674548 38005525

[35] Logeswaran, J. & Rani, R. Design and analysis of S-shaped broadside coupled metamaterial unit cell as a sensor to ease the classification of different oil samples. *Progress Electromagn. Res. Lett.* **110**, 83–91 (2023). 10.2528/PIERL23012501

